# María Teresa Miras Portugal: a pioneer in the study of purinoceptors in chromaffin cells

**DOI:** 10.1007/s11302-023-09934-1

**Published:** 2023-03-21

**Authors:** Antonio R. Artalejo, Marina Arribas-Blázquez, María Victoria Barahona, Celia Llorente-Sáez, Luis Alcides Olivos-Oré

**Affiliations:** Departament of Pharmacology and Toxicology, Faculty of Veterinary Medicine, Universidad Complutense de Madrid, Avda. Puerta de Hierro s/n, 28040 Madrid, Spain

**Keywords:** Purinoceptors, Purinergic signalling, Chromaffin cells, Adrenal medulla, Diadenosine polyphosphates

## Abstract

María Teresa Miras Portugal devoted most of her scientific life to the study of purinergic signalling. In an important part of her work, she used a model system: the chromaffin cells of the adrenal medulla. It was in these cells that she identified diadenosine polyphosphates, from which she proceeded to the study of adrenomedullary purinome: nucleotide synthesis and degradation, adenosine transport, nucleotide uptake into chromaffin granules, exocytotic release of nucleotides and autocrine regulation of chromaffin cell function via purinoceptors. This short review will focus on the current state of knowledge of the purinoceptors of adrenal chromaffin cells, a subject to which María Teresa made seminal contributions and which she continued to study until the end of her scientific life.

## In the beginning was dopamine beta-hydroxylase

María Teresa Miras Portugal began her scientific career by studying the properties of dopamine beta-hydroxylase (DBH) from adrenal chromaffin cells of the bovine adrenal medulla [1]. This is an enzyme located in both the soluble and membrane-bound fractions of chromaffin granules responsible for the final step of norepinephrine biosynthesis. The abundance of these granules (10,000–30,000 granules per cell) made the adrenal medulla a privileged tissue for the isolation and characterisation of the enzyme. In a series of articles with Dominique Aunis and Paul Mandel, carried out at the Institute of Neurochemistry in Strasbourg, where she did her doctoral thesis, she isolated the enzyme, characterised it structurally and kinetically [2, 3], and described its inhibition by 6-hydroxy-dopamine [4]. Back in Spain, María Teresa also undertook to characterise human DBH present in serum [5, 6] and to study its modifications in stress conditions such as hypoglycaemia and cold [7] or in streptozotocin-induced diabetes [8, 9]. The fact that this enzyme is released into the bloodstream together with catecholamines was one of the first biochemical proofs that this release takes place by the mechanism of exocytosis [10] and led at that time to investigate whether circulating levels of the enzyme were a good indicator of the activity of the adrenal medulla and peripheral adrenergic nerves [11–13].

## From DBH to nucleotides

In addition to catecholamines and DBH, chromaffin granules also contain chromogranins and many different nucleotidic compounds. It was from the DBH of the chromaffin cells that María Teresa came into the purinergic field. Her initial interest was in purine metabolism, which led to the characterisation of adenine phosphoribosyltransferase, adenosine kinase [14, 15], and the study of adenosine transport including its positive modulation by ATP [16–21]. These studies coincided with the description of the presence of diadenosine polyphosphates (Ap_4_A, Ap_5_A) in the chromaffin granules, from where they are released together with the classical nucleotides (ATP, ADP) and the other components of the “secretory cocktail” [22, 23].

## From nucleotides to purinoceptors

Traditionally, the high concentration of ATP in granules was thought to be due to its colligative properties, allowing the storage of large amounts of catecholamines with a limited increase in osmolarity [24]. However, ATP and the other purinergic nucleotides are not limited to this role; once released together with catecholamines, they contribute to the autocrine and paracrine regulation of the function of chromaffin cells and neighbouring endothelial cells through many different purinergic mechanisms and receptors. María Teresa was also a pioneer in the identification of these receptors, and although she gradually moved to the study of purinergic signalling in the central nervous system, both in physiological and pathological conditions, she never completely abandoned the adrenal medulla. She was not alone in this work, since one of her outstanding strengths was to surround herself with very capable collaborator—Magdalena Torres, Esmerilda G. Delicado, Enrique Castro, Jesús Pintor, Raquel Pérez-Sen—and the ability to establish scientific collaborations with other research groups. The first two articles in this line of research showed, on the one hand, the ability of cholinergic stimulation to induce the release of Ap_4_A and Ap_5_A from chromaffin cell cultures and perfused bovine adrenal glands, in a ratio vs ATP similar to that found in chromaffin granules [25] and, on the other hand, the effects of these compounds on the secretion of catecholamines by chromaffin cells [26]. In this respect, diadenosine polyphosphates (Ap_3_A, Ap_4_A and Ap_5_A) increase the basal release of catecholamines, this effect being strictly dependent on the presence of Ca^2+^ in the extracellular medium; they also inhibit the release of catecholamines induced by nicotinic stimulation and exert a dual stimulatory (Ap_3_A, Ap_4_A) or inhibitory (Ap_5_A) effect on the release induced by high extracellular K^+^. These two papers were quickly followed by another paper on Ap_6_A with similar findings [27].

The results published in these articles paved the way for the characterisation of the signalling mechanisms and purinergic receptors involved in the effects of diadenosine polyphosphates. Using Ca^2+^ microfluorometry with the fura-2 probe, it was shown that Ap_4_A and Ap_5_A increase the cytosolic Ca^2+^ concentration ([Ca^2+^]_i_) by promoting its release from intracellular pools. These effects would be mediated by a metabotropic P2Y receptor, since they were sensitive to depletion of internal Ca^2+^ stores by pretreatment with bradykinin or removal of extracellular Ca^2+^, mimicked and desensitised by the agonist adenosine 5′-O-(2-thiodiphosphate), and blocked by the P2Y receptor antagonist cibachrome blue [28]. The same P2Y receptor would also be responsible for the inhibition of Ca^2+^ influx induced by nicotinic stimulation (see mechanism of action below), leading to a reduction in catecholamine secretion, and the inhibition of adenosine transport by Ap_4_A, an effect that involves the activation of protein kinase C [28, 29].

A more detailed characterisation of purinergic receptors in bovine chromaffin cells was undertaken in collaboration with the group of Dr. Luis Rosário at the University of Coimbra. The results obtained by single cell microfluorometry of ([Ca^2+^]_i_ showed a heterogeneous distribution of purinoceptors in bovine chromaffin cells, as well as a clear dependence on Ca^2+^ influx of ATP-induced ([Ca^2+^]_i_ increase. The fact that ATP-sensitive cells also responded to 2-methylthioadenosine triphosphate (2MeSATP) and UTP with increases in [Ca^2+^]_i_ was consistent with the expression of both P2X and P2Y receptors [30]. Likewise, bovine adrenal endothelial cells expressed a P2Y-type receptor coupled to Ca^2+^ mobilisation from intracellular pools. P2X receptor expression was confirmed in subsequent work by blocking ATP-induced [Ca^2+^]_i_ elevations in UTP-insensitive cells with suramin; furthermore, in another subset of cells, ATP and UTP induced [Ca^2+^]_i_ elevations that were insensitive to extracellular Ca^2+^ depletion and suramin, confirming P2Y receptor expression in bovine chromaffin cells. Notably, P2X receptor activation is coupled to preferential norepinephrine secretion, whereas P2Y receptor activation by itself does not induce a secretory response [31]; for a confirmation and extension of these studies, see [32, 33].

Coinciding with or immediately following the publication of this work, other research groups joined in the characterisation of purinergic receptors in chromaffin cells of several species (e.g. bovine, rat, guinea pig) [34]. In addition, the use of electrophysiological techniques allowed progress to be made in the study of P2X receptors, the modulation of voltage-gated Ca^2+^ channels by P2Y receptors and the effect of purinergic agonists on stimulated exocytosis through changes in cell capacitance.

In bovine chromaffin cells, ATP application reduces the Ca^2+^ current by inhibiting voltage-dependent Ca^2+^ channels (N, L, and P/Q-types). The inhibition is partially voltage-dependent (reversed by membrane depolarisation) and is mediated by a pertussis toxin (PTX)-sensitive G protein [35], suggesting the involvement of a P2Y receptor. This effect results in inhibition of exocytosis [36–41], providing a mechanism for the inhibition of catecholamine release previously described for purinergic agonists, including the diadenosine polyphosphates [26]. Of the various P2Y receptors expressed in chromaffin cells (P2Y_1_, P2Y_2_, P2Y_4_, P2Y_6_, P2Y_12_, P2Y_13_, and P2Y_14_) [42], the P2Y_12_ receptor mediates the effects on both voltage-gated Ca^2+^ channels and the secretory response [43]. The use of carbon fibre amperometry to record catecholamine release from individual vesicles showed that P2Y_12_ purinergic modulation reduces not only the probability of vesicle release but also the quantal content or amount of catecholamines released from each vesicle. This is a G_i_*βγ* protein-mediated effect that influences the opening time of the vesicle fusion pore, reducing it [44]. Notably, this effect is also voltage-dependent, such that membrane depolarisation reduces the effect on quantal size [42].

Few studies have investigated the expression of P2X receptors in chromaffin cells using electrophysiological techniques. For example, Otsuguro et al. [45] reported that ATP evoked an inward current in voltage-clamped guinea pig chromaffin cells with a reversal potential of approximately 0 mV, suggesting a non-selective cation permeability of the ATP-gated channel. As already mentioned for bovine chromaffin cells, ATP stimulation is coupled to catecholamine secretion and an increase in [Ca^2+^]_i_, both of which are suppressed by removal of extracellular Ca^2+^ [46]. These results were extended by Liu et al [47] who concluded that P2X receptors in guinea pig chromaffin cells share many characteristics of the P2X2 receptor subtype. Interestingly, rat chromaffin cells barely express P2X receptors under normal conditions. However, chromaffin cells from animals experiencing neuropathic pain, due to chronic sciatic nerve constriction, overexpress functional P2X3 and P2X7 receptors, which would be activated by released adenine nucleotides to increase cellular excitability (P2X3 receptor) or directly induce exocytosis (P2X7 receptor) [48, 49]. This unique example of purinoceptor plasticity in chromaffin cells was María Teresa’s last contribution to the purinergic field in this neuroendocrine cell, a cell model she used throughout her scientific career.

## Concluding remarks

María Teresa soon came across the chromaffin cells of the adrenal medulla. Knowing their ability to release large quantities of catecholamines into the bloodstream, which, like Don Quixote, go on all sorts of adventures throughout the body, she soon realised that they had a faithful squire, adenine nucleotides, which, like Sancho, were responsible for ensuring good accommodation in the chromaffin granules and regulating their raids into the extracellular medium. María Teresa was able to extend many of her findings on chromaffin cells to the neurons of the central nervous system, both in physiological and pathological conditions (Alzheimer’s disease, Huntington’s disease, epilepsy, etc.), but she never completely abandoned the adrenal medulla. The authors of this article had the good fortune to accompany María Teresa on some of her scientific adventures. Let this account serve as a memorial and a tribute to her.
